# 

**Published:** 1897-05

**Authors:** 


### PERSONAL.

Drs. John W. Adams, of Philadelphia; H. P. Eves, of Wilmington, Del.; and W. Horace Hoskins, of Philadelphia, will act as veterinary inspectors at the coming Philadelphia horse-show.

Dr. Charles Williams, of Philadelphia, was fined twenty dollars cash and costs by the authorities of Montgomery County for docking the tails of a pair of ponies.

Dr. Harry Walters, of Wilkesbarre, is lecturing on veterinary dentistry at the Veterinary Department of the University of Pennsylvania.

Dr. Herbert Neher recently addressed the New York Microscopical Society on fatty or adipose tissue.

Prof. A. Liautard sailed for Europe February 8th, to join his wife and daughter.

Prof. Schwarzkopf, of Chicago, has located at Flushing, L. I., succeeding to the practice of the late Dr. W. T. Simmons.

Dr. James A. Marshall, of Philadelphia, has been elected President of the Belmont Driving Club.

Dr. Herbert Neher, of New York City, has been elected professor of clinical medicine of the American Veterinary College by the Board of Trustees.

Dr. H. T. George has succeeded as meat-inspector for Camden, N. J., Dr. W. B. E. Miller, who has held the position for several years at stated intervals.

Prof. A. H. Baker, of the Chicago Veterinary College, was one of the speakers at the Cook County (Ill.) Farmers' Institute.

Dr. Edward P. Dowd, of the Bureau of Animal Industry, has been transferred from Philadelphia to Boston, Mass.

Dr. E. H. Shepard, of Cleveland, Ohio, is now recovering from a long and serious illness.

Dr. C. H. Doepel, recently assistant to Dr. E. B. Ackerman, has changed his vocation and joined his father in the meat business.

Dr. William Kaul, of St. Mary's, Pa., is associated with his father in the Elk Stock Farm.

Dr. Charles Schaufler, of Philadelphia, holds the important position of assistant general manager of the Union Traction Company.

Drs. E. C. Switzer and F. G. Scannell, former students of the United States Veterinary College, finished their course at the Veterinary Department of the Columbian University.

One of the graduating class of the American Veterinary College was a graduate of the Chicago Veterinary College.

Dr. A. W. Clement, of Baltimore, has been selected veterinarian to the Elkridge Fox-hunting Club Show.

Dr. Charles Lovejoy, of Princeton, Ill., has been appointed State Veterinarian by Governor Tanner, to succeed Dr. M. R. Trumbower. Dr. Lovejoy is not a graduate of any veterinary college.

Assistant Secretary of Agriculture, Dr. Charles Dabney, has been placed in charge of all the scientific work of the Department of Agriculture.

Veterinarians Olof Schwarzkopf and F. T. McMahon are enthusiastic members of the Chicago Equestrian Club.

Dr. M. J. Dunn, of Detroit, is President of the Michigan State Protective Horseshoers' Union.

Dr. Charles Bridge, of Philadelphia, met with a serious accident by falling from a trolley car, sustaining a concussion of the brain.

President McKinley has recently provided himself with a Kentucky saddle-gaited horse. Mayor Harrison, of Chicago, will follow his father's footsteps as Mayor in ranging over the municipal domains of his city on a gaited saddle-horse.

